# Urinary cytokines in *Schistosoma haematobium*-infected schoolchildren from Tana Delta District of Kenya

**DOI:** 10.1186/1471-2334-14-501

**Published:** 2014-09-15

**Authors:** Kariuki H Njaanake, Paul E Simonsen, Birgitte J Vennervald, Dunstan A Mukoko, Claus M Reimert, Kimani Gachuhi, Walter G Jaoko, Benson B Estambale

**Affiliations:** Department of Medical Microbiology, College of Health Sciences, University of Nairobi, P.O. Box 19676 – 00202, Nairobi, Kenya; Section for Parasitology and Aquatic Diseases, Faculty of Health and Medical Sciences, University of Copenhagen, Dyrlægevej 100, 1870 Frederiksberg C, Denmark; Division of Vector Borne & Neglected Tropical Diseases, Ministry of Public Health & Sanitation, P.O. Box 54840–00202, Nairobi, Kenya; Centre for Biotechnology Research & Development, Kenya Medical Research Institute, P.O. Box 54840–00200, Nairobi, Kenya; Jaramogi Oginga Odinga University of Science and Technology, P.O. Box 210–40601, Bondo, Kenya

## Abstract

**Background:**

Pathological changes due to infection with *Schistosoma haematobium* include cytokine-mediated urinary tract inflammation. The involved cytokines may be excreted in urine and their presence in urine may therefore reflect *S. haematobium*-related urinary tract pathology. The present study, for the first time, reports on the relationship between selected cytokines in urine and infection with *S. haematobium* in children from an area highly affected by this parasite.

**Methods:**

Children aged 5–12 years from two primary schools in Tana Delta District of Kenya were examined for *S. haematobium* eggs using urine filtration technique, for haematuria using dipstix and for eosinophil cationic protein (ECP), IL-6, IFN- γ, TNF-α and IL-10 levels using ELISA, and for *S. haematobium*-related urinary tract pathology using ultrasonography. In addition, venous blood was examined for serum IL-6, IFN- γ, TNF-α and IL-10 levels using ELISA.

**Results:**

There was no significant correlation between urinary and serum levels of IL-6, IFN- γ, TNF-α or IL-10. There was no significant difference in geometric mean intensity (GMI) in any of the serum cytokines, or in urinary TNF-α or IFN-γ, between children with light and heavy *S. haematobium* infections. However, children with heavy *S. haematobium* infections had significantly higher GMI of urinary IL-6 (*p* < 0.001) and lower GMI of urinary IL-10 (*p* = 0.002) than children with light infections. There was also a significant positive correlation between urinary IL-6 and urinary ECP (p < 0.001) and a significant negative correlation between urinary IL-10 and urinary ECP (*p* = 0.012).

**Conclusion:**

Urinary IL-6 was positively correlated to and IL-10 was negatively correlated to infection intensity and urinary tract inflammation in *S. haematobium*-infected children. Urinary IL-6 and IL-10 ELISA may be a useful non-invasive tool to complement the already available tools for studying *S. haematobium*-related urinary tract pathology in children.

**Electronic supplementary material:**

The online version of this article (doi:10.1186/1471-2334-14-501) contains supplementary material, which is available to authorized users.

## Background

*Schistosoma haematobium*, the causative agent of urinary schistosomiasis, infects over 112 million individuals and results in over 150,000 deaths annually in sub-Saharan Africa [1]. In chronic *S. haematobium* infections, eggs lodged in the urinary tract elicit immune responses which result in pathology mainly characterized by inflammatory cell infiltration, granuloma formation, urinary tract fibrosis and renal dysfunction [2, 3]. Most of the available information on pathogenesis in infected individuals is a result of studies using markers of morbidity such as egg counts, urinary eosinophil cationic protein (ECP), ultrasound and immunological proxies of inflammation [4]. However, these markers have inherent limitations and there is need for more tools for use in the study of *S. haematobium*-related pathology in infected individuals [4].

It has been shown that a strong pro-inflammatory tumour necrosis factor (TNF)-α response relative to the anti-inflammatory interleukin (IL)-10 response is associated with increased ultrasound-detectable pathology in the urinary tracts of *S. haematobium*-infected children [5]. However, there is no clear consensus on the association between the patterns of cytokine response and *S. haematobium* infections. For example, studies using peripheral blood mononuclear cell culture demonstrated a positive association between Th1 cytokines, such as TNF-α and urinary tract pathology in infected individuals [5, 6]. However, another study, using whole blood cultures from infected individuals, could not demonstrate a significant association between Th_1_ or Th_2_ cytokines and *S. haematobium* infection status or intensity [7].

At least one reason may be advanced to explain the observed variability of cytokine patterns in relation to schistosome-related pathology. For example, some of the studies have concentrated on systemic cytokine responses and related them to local inflammatory processes in the urinary tract tissue instead of studying cytokine responses directly at the affected organ. The interpretation of results may therefore be complicated by the fact that immune cells may secrete different cytokines in different environments and due to different stimuli [8]. Furthermore, most of the cytokines involved in local inflammatory processes are secreted not only by immune cells but also by non-haematopoietic cells [9–13].

Studies on bacterial infections in the urinary tract have demonstrated that there is substantial cytokine secretion from the mucosa, indicating that the urinary tract mucosa is an immunologically active tissue. For example, it has been possible to induce *in vivo* secretion of measurable levels of IL-6 in urine from urinary tract mucosa of volunteers whose urinary tracts had been colonized with *Escherichia coli*[14]. In a study using epithelial cells from the human urinary tract, *E. coli* elicited high levels of IL-6 but not TNF-α whereas human peripheral blood monocytes secreted both IL-6 and TNF-α as well as other cytokines in response to *E. coli*[15]. This suggests that there could be differences between systemic and local mucosa cytokine responses during infections in the urinary tract.

A study of local cytokine responses in the urinary tract mucosa may yield important information about their role in the pathogenesis in the affected organs during *S. haematobium* infection. No studies have so far been reported on the relationship between *S. haematobium*-related urinary tract pathology and urinary cytokines despite the importance of the pathological mucosal inflammation in infections with this parasite. The present study examined the relationship between selected urinary and serum cytokines [IL-6, interferon-γ (IFN-γ), TNF-α, and IL-10], urinary ECP, and *S. haematobium* infection and morbidity in children from two village primary schools in Tana Delta District of Kenya.

## Methods

### Study area and study design

The cross-sectional study was carried out in Tana Delta District, northern coastal Kenya. Children aged 5 –12 years were recruited into the study from local primary schools in two villages (Kau and Ozi) located along the Tana River with a distance of about 5 km between them. One urine sample was collected from each child between 10:30 am and 12:00 pm on each of three consecutive days and examined for *S. haematobium* eggs. Urine samples were analyzed for cytokines and eosinophil cationic protein (ECP). One venous blood sample was collected from each child and analyzed for serum cytokines. The urinary tract of each child was examined by ultrasonography for *S. haematobium* infection-related pathology.

Ethical review and approval to carry out the study was obtained from the Kenyatta National Hospital/ University of Nairobi Ethics and Research Committee in Kenya (Ethical approval No. P91/3/2009). For each child’s participation, a written informed consent was obtained from one of the parents. All children were treated with praziquantel (40 mg/kg body weight) at the end of the study.

### Urine examination for *S. haematobium*eggs

Ten milliliter of each of the three consecutive urine samples from each child was filtered through a polycarbonate filter (12 μm pore-size; GE Water & Process Technologies Inc., USA) and the filter was examined microscopically for *S. haematobium* eggs [16]. The results, based on arithmetic mean of the three egg counts from each child, were classified into negative, light (1–49 eggs/10 ml urine) or heavy infections (≥50 eggs/10 ml urine) according to WHO [17, 18].

### Urinary and serum cytokine ELISA

Aliquots of well suspended urine samples were collected from the children for cytokine and ECP analysis. The samples were kept cold in a cool box during transport to the laboratory where they were frozen at – 20°C within 4 hrs after collection and until used for cytokine or ECP analysis. One venous blood sample (2 ml) was collected, in plain Nunc tubes, from each child in the field and allowed to clot at room temperature for 30 minutes. It was then transported to the laboratory within 4 hrs after collection, in a cool box, where it was centrifuged at 1,000 × g for 10 minutes to remove the clot. The resultant serum was transferred to a new Nunc tube and frozen at – 20°C until use in cytokine ELISA.

Serum and non-filtered urine samples were thawed, re-suspended and part of each sample (100 μl) was analysed for the concentrations of IL-6, IL-10, TNF-α and IFN-γ through solid phase sandwich ELISA using the BD OptEIA^TM^ ELISA Kit II format (BD Biosciences, United States), with a lower detection limit of 0.01 pg/ml, according to the manufacturer’s instructions. The assays were performed at room temperature and both undiluted serum and urine samples were treated in a similar way. A single test was run per sample and the ELISA plates were read at 450 nm within 30 minutes and the average of the two readings for each child calculated. Blood cell counts were performed on the children’s blood samples (data not shown) and none had neutrophilia, a marker of bacterial infections. It was therefore assumed that the measured urinary cytokine levels were mainly due to infections with *S. haematobium*.

### Urinary ECP ELISA

Extraction of urine samples for and measurement of urinary ECP was carried out as previously explained in detail [19]. Briefly, one volume of well-suspended urine was mixed with 1 volume of extraction buffer (1% N-cetyl-N,N,N-trimethyl-ammonium bromide [CTAB] in 0.15 M NaCl). After 1 cycle of freeze-thawing, the sample was mixed on a vortex-mixer and centrifuged for 10 min at 3,000 X g at 4°C. The supernatants containing the extracted proteins were removed and used for ECP determinations by a polyclonal sandwich type ELISA using the biotin-avidin-peroxidase amplification step which measures ECP in the range of 15–1,000 pg/ml. ECP purified from extracts of human blood eosinophils was used as standards. Before measurement, the standard and urine extracts were diluted by adding 200 μl of standard or urine extract to 800 μl of sample buffer (0.1% Tween 20, 0.1% CTAB, 20 mM EDTA, 0.2% human serum albumin, and 0.1% NaN3 in phosphate-buffered saline, pH 7.4). Reading of the ECP concentrations was done using Reading Microplate Manager 4.0® (Bio-Rad Laboratories, Inc.) software at a measurement wavelength of 490 nm and reference wavelength of 595 nm. Samples were analysed in duplicates and the average of the two readings for each child calculated.

### Urinary tract ultrasound examination

Ultrasound examination of the urinary tracts of each child was performed by an experienced ultrasonographer using a portable convex sector scanner (SSD-500®; *Aloka*, Tokyo, Japan) according to the Niamey protocol [20] and bladder, ureter and kidney pathology was recorded. If any kidney and/ or ureter dilatation was observed the child was asked to empty the bladder and come back for re-examination.

### Test for haematuria

Urine samples were examined visually for presence or absence of blood and using dipstix (URISCAN®, YD Diagnostics, Korea) according to the manufacturer’s instructions for occult blood (microhaematuria). Trace haematuria was regarded as negative and anything above trace was regarded as positive for microhaematuria.

### Statistical analysis

Data were analysed statistically using Stata (Version 12). Geometric mean intensities (GMI) were calculated for non-normally distributed continuous variables such as *S. haematobium* egg counts and cytokine levels using the formula: antilog10 [(Σlog10 x + 1)/n)]-1, with n being the number of children examined. GMI were compared between groups by using t-test on log(x + 1)-transformed values. Standard errors (S.E.) on GMI were calculated as: antilog [mean of log-transformed (x + 1) ± S.E. on log transformed (x + 1)]-1. Proportions such as prevalence were compared between groups using Pearson χ^2^ test. The linear association between continuous variables was assessed using Pearson correlation analysis. *p*-values less than 0.05 were considered significant in all tests.

## Results

### Study population

One hundred and fifty-eight (158) children, 71 boys and 87 girls, participated in the study. The mean age of the children was 9.8 years but boys were significantly older than girls (10.1 and 9.5 years old, respectively, *p* = 0.039). The overall prevalence of *S. haematobium* infections based on microscopic examination of three urine samples from each child was 94.3%. There was no significant difference in prevalence of infections between boys and girls (96.8% and 93.1%, respectively, *p* = 0.52). The overall GMI was 64.8 eggs/ 10 ml urine but boys had significantly higher GMI than girls (91.9 and 48.6 eggs/10 ml urine, respectively, *p* = 0.049).

As the area is highly prevalent for *S. haematobium* and the sensitivity of the urine filtration technique is relatively low in individuals with low intensities [18, 21] it was assumed that the 9 egg-negative children were also infected but that the eggs were missed during microscopy. The egg-negative children were thus grouped together with those with light infections in the following analyses.

### Relationship between urinary and serum cytokine levels

**Figure 1:**
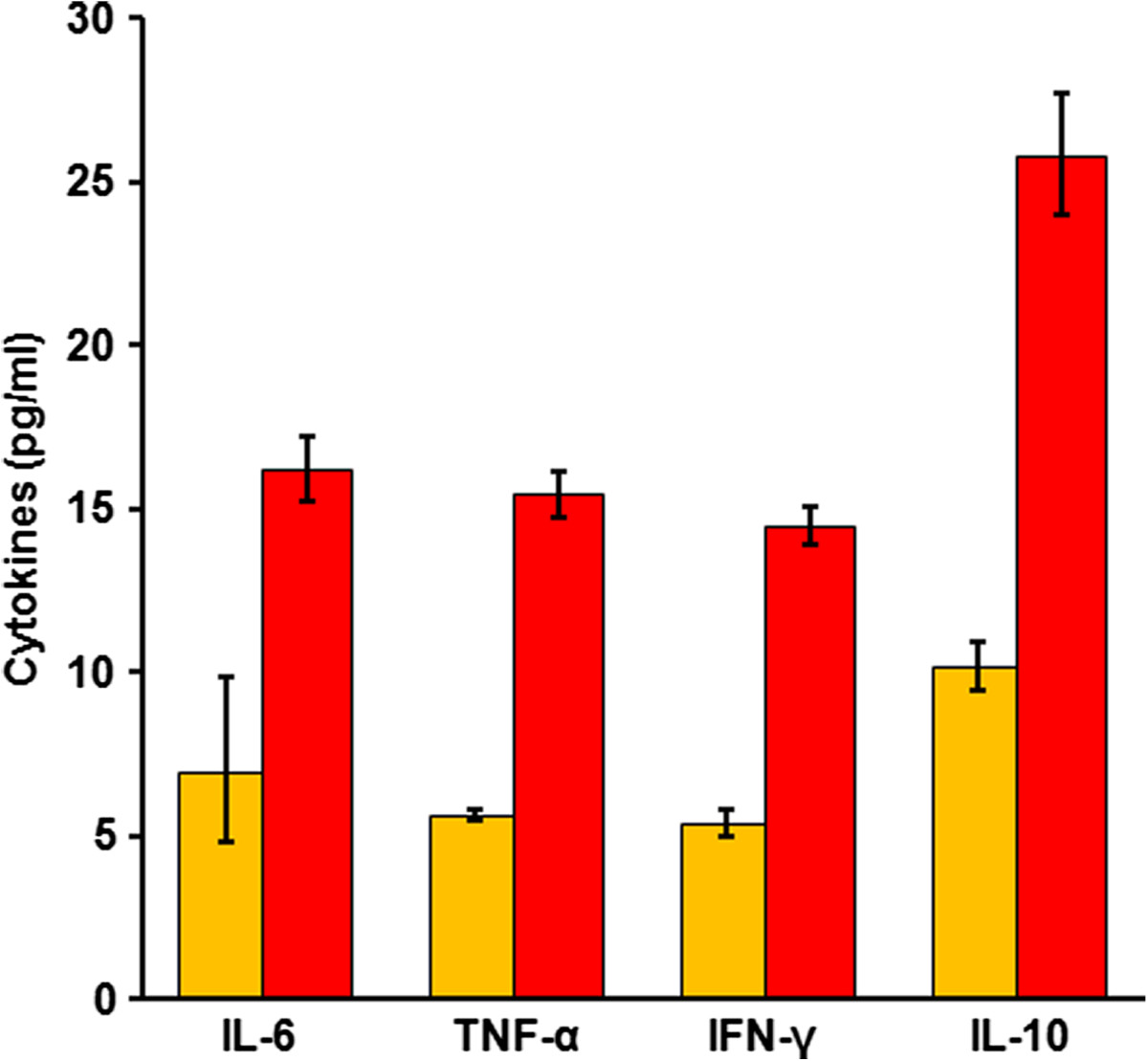
**GMI of urinary and serum IL-6, TNF-α, IFN-γ and IL-10.** n = 158. Orange bars = Urinary cytokines; Red bars = Serum cytokines. Vertical lines indicate standard errors (±S.E.).

The urine and serum samples from 158 children were analysed for levels of IL-6, TNF-α, IFN-γ and IL-10, and the geometric mean intensities (GMI) are shown in Figure 1. Considering all 158 children, the GMI of serum cytokines were higher than those of urinary cytokines.

The correlation between urinary and serum cytokines was assessed for all 158 children using correlation analysis. Overall, there was no significant correlation between levels of urinary and serum IL-6 (r = -0.01, *p* = 0.87), TNF-α (r = -0.04, *p* = 0.59), IFN-γ (r = -0.05, *p* = 0.50) or IL-10 (r = -0.03, *p* = 0.70).

For serum TNF-α, IFN-γ and IL-10 there were 40, 39 and 27 children, respectively, who had levels below the lower detection limit of the test. For each of the cytokines, these children were not significantly different from the rest of the children in the study group with regard to age, sex or *S. haematobium* infection intensity (data not shown). They were regarded as non-responders for the respective serum cytokines, and since they could negatively skew the distribution of serum cytokine data, they were excluded in the subsequent analyses of the respective cytokines. However, all 158 children had detectable levels of urinary cytokines.

### Relationship between cytokines and *S. haematobium*infection intensity

**Figure 2:**
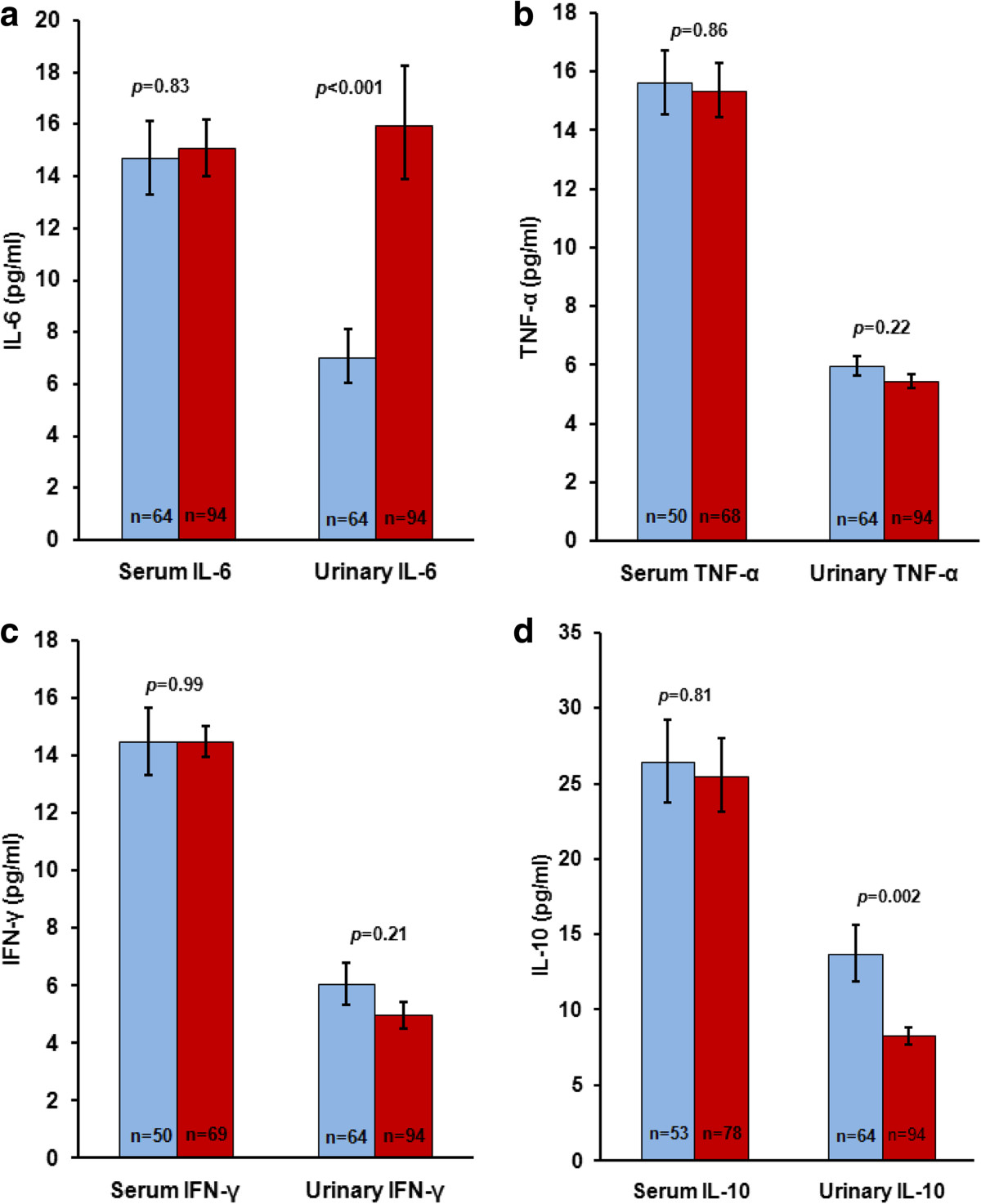
**GMI of urinary and serum IL-6 (a), TNF-α (b), IFN-γ (c) and IL-10 (d) in relation to** ***S. haematobium*** **infection intensity.** *p-*values above the bars indicate significance level of differences between light and heavy *S. haematobium* infection intensity groups. Blue bars = Light infection; Red bars = Heavy infection. n = number of children in group. Vertical lines indicate standard errors (±S.E.).

The GMI of urinary and serum IL-6, TNF-α, IFN- γ and IL-10 in relation to *S. haematobium* infection intensity are shown in Figures 2a,b,c and d, respectively. There were no significant differences in GMI of serum IL-6, TNF-α, IFN-γ or IL-10 between children with light and children with heavy *S. haematobium* infections. Similarly, there were no significant differences in GMI of urinary TNF-α or IFN-γ between children with light and heavy *S. haematobium* infections. Considering all 158 children, those with light *S. haematobium* infections however had significantly lower GMI of urinary IL-6 and significantly higher GMI of urinary IL-10 than children with heavy *S. haematobium* infections (*p* < 0.001 and *p* = 0.002, respectively). The results remained significant when the 9 *S. haematobium* egg-negative children were excluded from this analysis, with children with light *S. haematobium* infections having significantly lower GMI of urinary IL-6 and significantly higher GMI of urinary IL-10 than those with heavy *S. haematobium* infections (*p* < 0.001 and *p* = 0.007, respectively).

### Relationship between cytokines, urinary tract pathology and morbidity markers

Eight children did not return for ultrasound examination after voiding their bladders and were therefore excluded in the analyses of ultrasound detectable pathology. Overall, urinary tract pathology was reported in 62 (41%) children. Lower urinary tract pathology was reported in 52 (35%) of the children whereas upper urinary tract pathology was reported in 17 (11%) of the children, which had mean ages of 10.2 years and 10.2 years, respectively. Seven of the children had both lower and upper urinary tract pathology.

There were no significant differences in GMI of urinary IFN-γ or IL-10 between children with and those without urinary tract pathology (*p* = 0.81 and 0.19, respectively). Children with urinary tract pathology, however, had significantly higher GMI of urinary IL-6 and significantly lower GMI of urinary TNF-α than those without (*p* = 0.016 and 0.015, respectively). There were no significant differences in GMI of serum IL-6, TNF-α, IFN-γ or IL-10 between children with and those without general urinary tract pathology (*p* = 0.43, 0.26, 0.33 and 0.23, respectively).

Children with haematuria had significantly higher GMI of urinary IL-6 (*p* < 0.001; n = 158) but lower GMI of urinary IL-10 (*p* = 0.005, n = 158) than children without haematuria. There was however no significant difference between children with haematuria and those without in GMI of urinary TNF-α (*p* = 0.11; n = 158) or IFN-γ (*p* = 0.29; n = 158). Similarly, there were no significant differences between children with haematuria and those without in GMI of serum IL-6 (*p* = 0.63; n = 158), TNF-α (*p* = 0.38; n = 118), IFN-γ (*p* = 0.34; n = 119) or IL-10 (*p* = 0.69; n = 131).

Data on urinary ECP from 4 children were not available and another 11 children had urinary ECP levels below the lower detection limit of the assay. However, there was no significant difference in serum cytokine levels between these 15 children and those whose urinary ECP data were available (data not shown). Considering the non-responders for the serum cytokines mentioned above, analyses of the relationship between serum cytokines and urinary ECP levels were only done for children who were responders for serum cytokines and had urinary ECP data available. These were 143 children for serum IL-6, 105 for TNF-α, 107 for IFN-γ and 118 for IL-10. Analyses of the relationship between urinary cytokines and urinary ECP levels were done for all 143 children whose urinary cytokine and ECP data were available. The linear association between urinary or serum cytokines and urinary ECP were assessed using correlation analysis for all children whose urinary ECP data were available.

**Figure 3:**
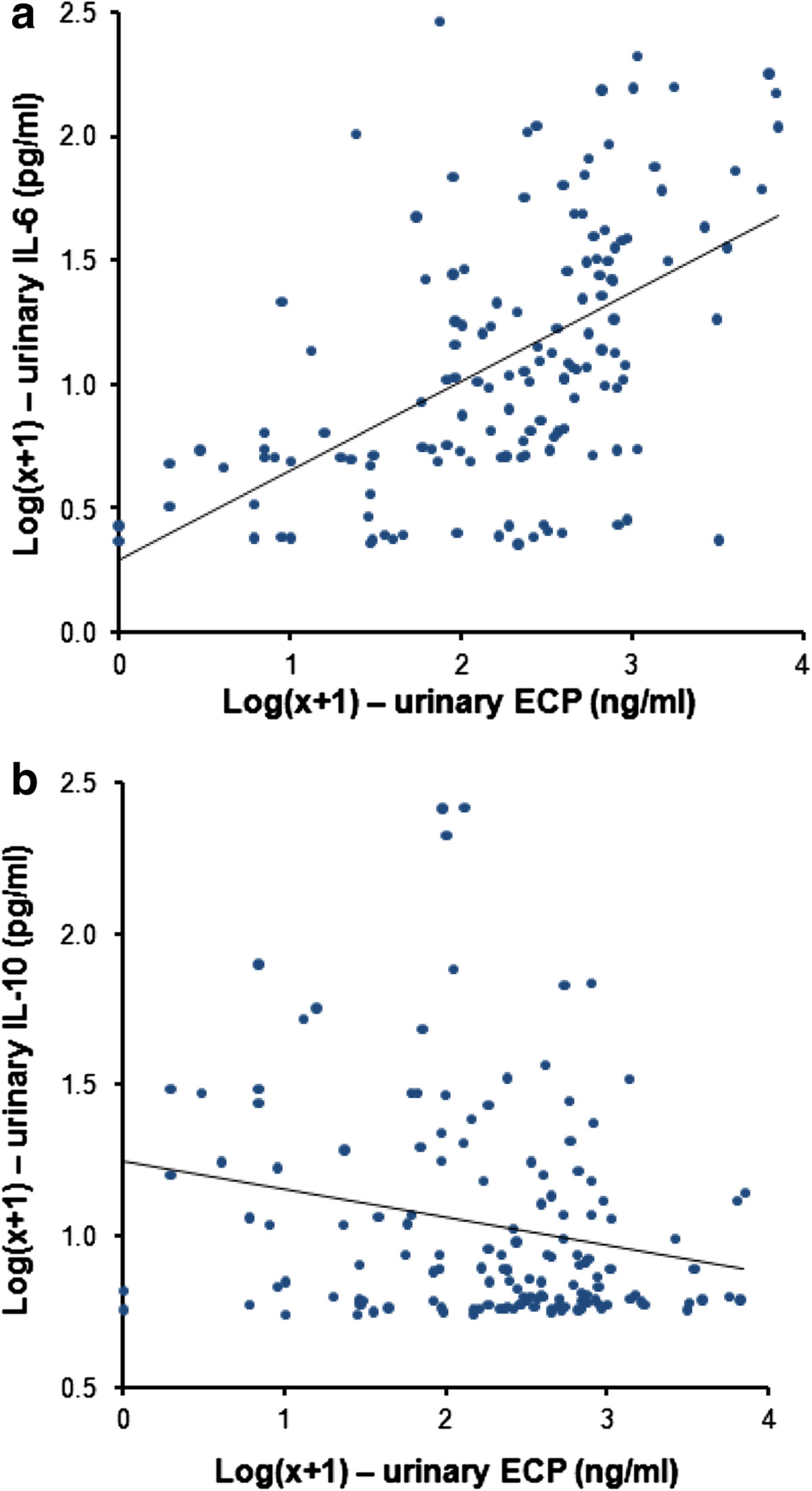
**Scatter diagram showing the association between urinary ECP and urinary IL-6 (a) and between urinary ECP and urinary Il-10 (b).** n = 143. The trend-lines shown are the linear regression lines.

There was a significant positive correlation between levels of urinary ECP and *S. haematobium* infection intensity (r = 0.23; *p* = 0.005; n = 143) but there was no significant correlation between levels of urinary ECP and serum IL-6 (r = -0.08, *p* = 0.32, n = 143), TNF-α (r = -0.11, *p* = 0.29; n = 105), IFN-γ (r = -0.11, *p* = 0.26; n = 107) or IL-10 (r = -0.13, *p* = 0.17; n = 118). There was a highly significant positive correlation between levels of urinary ECP and urinary IL-6 (r = 0.54, *p* < 0.001; n = 143) (Figure 3a) and a significant negative correlation between levels of urinary ECP and urinary IL-10 (r = -0.21, *p* = 0.012; n = 143) (Figure 3b). There was no significant correlation between levels of urinary ECP and TNF-α (r = -0.02, *p* = 0.80; n = 143) or IFN-γ (r = 0.02, *p* = 0.85; n = 143).

## Discussion

The present study reports for the first time the relationship between urinary cytokines IL-6, IFN-γ, TNF-α and IL-10, and *S. haematobium* infection and infection-associated urinary tract pathology in primary school children. Levels of urinary cytokine were lower than those of serum cytokines and there was no correlation between levels of urinary and serum IL-6, IFN-γ, TNF-α and IL-10 among the children. These findings concur with those of a previous study which demonstrated differences between urine and serum cytokine levels in children with bacterial infections of the urinary tract [22]. These findings suggest that there may be differences between systemic and local mucosal cytokine production or secretion during infections in the urinary tract.

A correlation between *ex vivo* cytokine secretion by antigen-stimulated peripheral blood cells and schistosome-related morbidity has previously been demonstrated both in studies on human schistosome infections and in *S. mansoni*-animal models where some features of the immune responses evoked in naturally infected humans are similar to those in controlled *S. mansoni*-animal model [5, 6, 23–25]. In contrast to findings from these studies, the present study showed no relationship between levels of serum IL-6, TNF-α, IFN-γ or IL-10 and markers of *S. haematobium*-related morbidity such as intensity of infection, haematuria or urinary ECP. There was no correlation between this pathology and levels of serum IL-6, TNF-α, IFN-γ or IL-10. It is noteworthy that the present study assessed levels of serum cytokines whereas previous studies have assessed *in vitro* cytokine production by *in vitro* cultured cells. For example, cytokine inhibitors are commonly present in sera of patients and may influence cytokine levels in serum samples as opposed to levels of *in vitro* produced cytokines [26]. The explanation for the differences may therefore, at least partially, be explained by differences between *in vivo* and *in vitro* conditions [27, 28].

Significantly higher levels of urinary IL-6 were observed in children with heavy *S. haematobium* infections, urinary tract pathology and haematuria than in children with light *S. haematobium* infections, and those without urinary tract pathology or haematuria. In addition, there was a significant positive correlation between levels of urinary IL-6 and urinary ECP. These findings suggest that the observed high levels of urinary IL-6 were as a result of local inflammation evoked by *S. haematobium* eggs in the urinary tract. Urinary IL-6 may have been produced by inflammtory cells such as eosinophils or by uroepithelial cells [15, 29] in schistosome egg-driven granulomas in the urinary tract whereafter it can be found in urine.

IL-10 is a potent anti-inflammatory cytokine produced by lymphocytes [27, 30, 31]. Several studies have demonstrated that IL-10 is involved in down-modulating pathological inflammatory responses in schistosomiasis [9, 24, 32]. Although levels of urinary IL-10 were significantly negatively correlated with levels of urinary ECP, infection intensity and haematuria, there was no significant difference in levels of urinary IL-10 between children with and without ultrasound-detectable urinary tract pathology. Ultrasound mainly detects late stage pathological changes characterised by fibrosis and calcification whereas urinary ECP is a marker of inflammation which characterises early pathology in the urinary tract during *S. haematobium* infections [4]. These results therefore suggest that urinary IL-10 is locally secreted and is mainly associated with early urinary tract pathology due to tissue-lodged *S. haematobium* eggs.

In a study, using cultures of peripheral blood mononuclear cells from infected children, it was demonstrated that TNF-α, a pro-inflammatory cytokine, was positively associated with *S. haematobium*-related bladder pathology [5]. In the present study, however, there was no significant difference in levels of urinary TNF-α between children with light and those with heavy *S. haematobium* infections or between those with and without haematuria although children without urinary tract pathology had significantly higher levels of urinary TNF-α than those with pathology. No immediate explanation could be advanced for this and further studies are needed to clarify this observation. In a recent mouse model study of urogenital schistosomiasis in which viable *S. haematobium* eggs were injected directly into the bladder wall thus recapitulating inflammatory cell activation and infiltration, urinary tract granuloma formation and fibrosis observed in infected humans, there was no significant changes in IFN-γ levels [33]. Although this study assessed systemic cytokines, the findings were in agreement with those of present study where there was no significant association between infection intensity or urinary tract pathology and IFN-γ levels hence no clear role of this cytokine in development of pathology. A recent study has shown that *S. haematobium*-infected children with bladder pathology had a significantly higher percentage of Th17 cells than those without pathology [34]. We do not know if urinary tract pathology was accompagnied by an increased level of IL-17 in serum or urine since Il-17 has not been measured in the current study.

## Conclusions

The present study is the first report on significant associations between urinary cytokine levels and *S. haematobium* infection and urinary tract pathology. Cytokines can be assessed non-invasively in urine samples and may potentially be important non-invasive tools in studying the inflammatory response leading to *S. haematobium*-related urinary tract pathology. This study may therefore provide an important scaffold for further studies on tissue inflammation and pathogenesis in *S. haematobium* infection and on how levels of local cytokine production change following treatment with praziquantel.

## Authors’ original submitted files for images

Below are the links to the authors’ original submitted files for images. Authors’ original file for figure 1 Authors’ original file for figure 2 Authors’ original file for figure 3
