# Supplementary figures and images for: Urinary cytokines in Schistosoma haematobium-infected schoolchildren from Tana Delta District of Kenya
Source: BMC Infect Dis. 2014 Sep 15;14:501. doi: 10.1186/1471-2334-14-501 (PMC4180153; doi:10.1186/1471-2334-14-501)

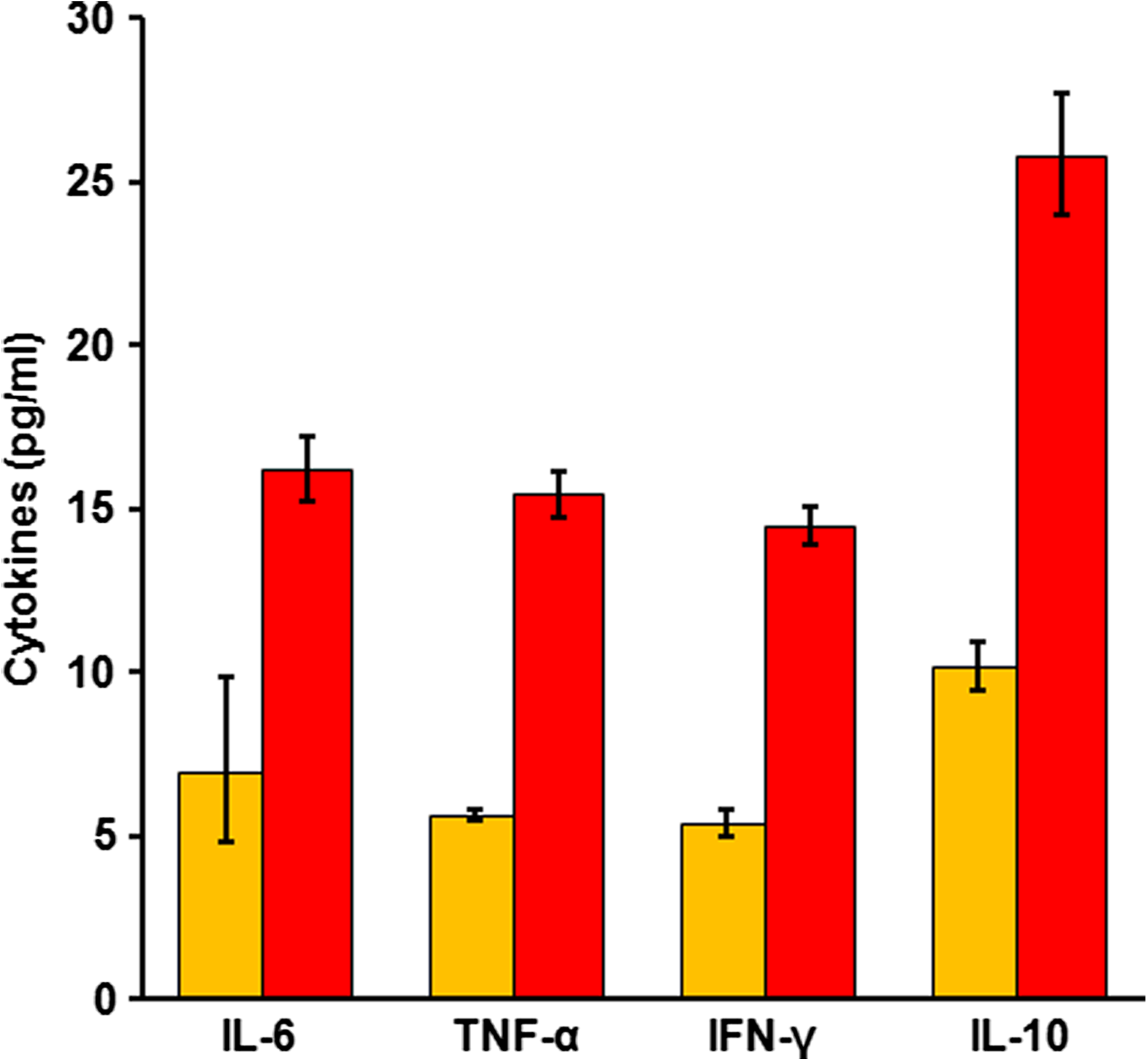

Supplement: Supplementary file 1 — Authors’ original file for figure 1 [file 12879_2014_3829_MOESM1_ESM.tif]

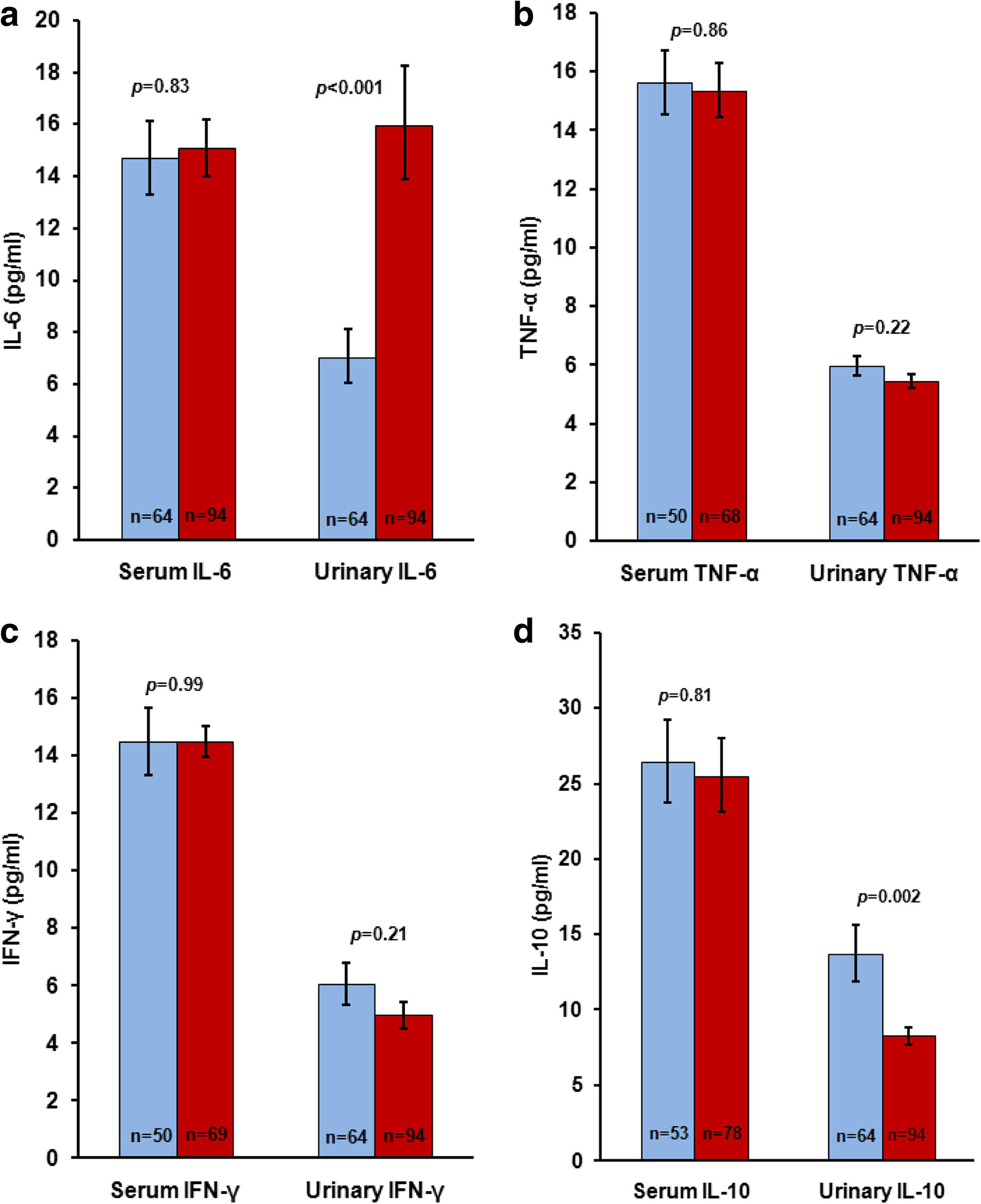

Supplement: Supplementary file 2 — Authors’ original file for figure 2 [file 12879_2014_3829_MOESM2_ESM.tif]

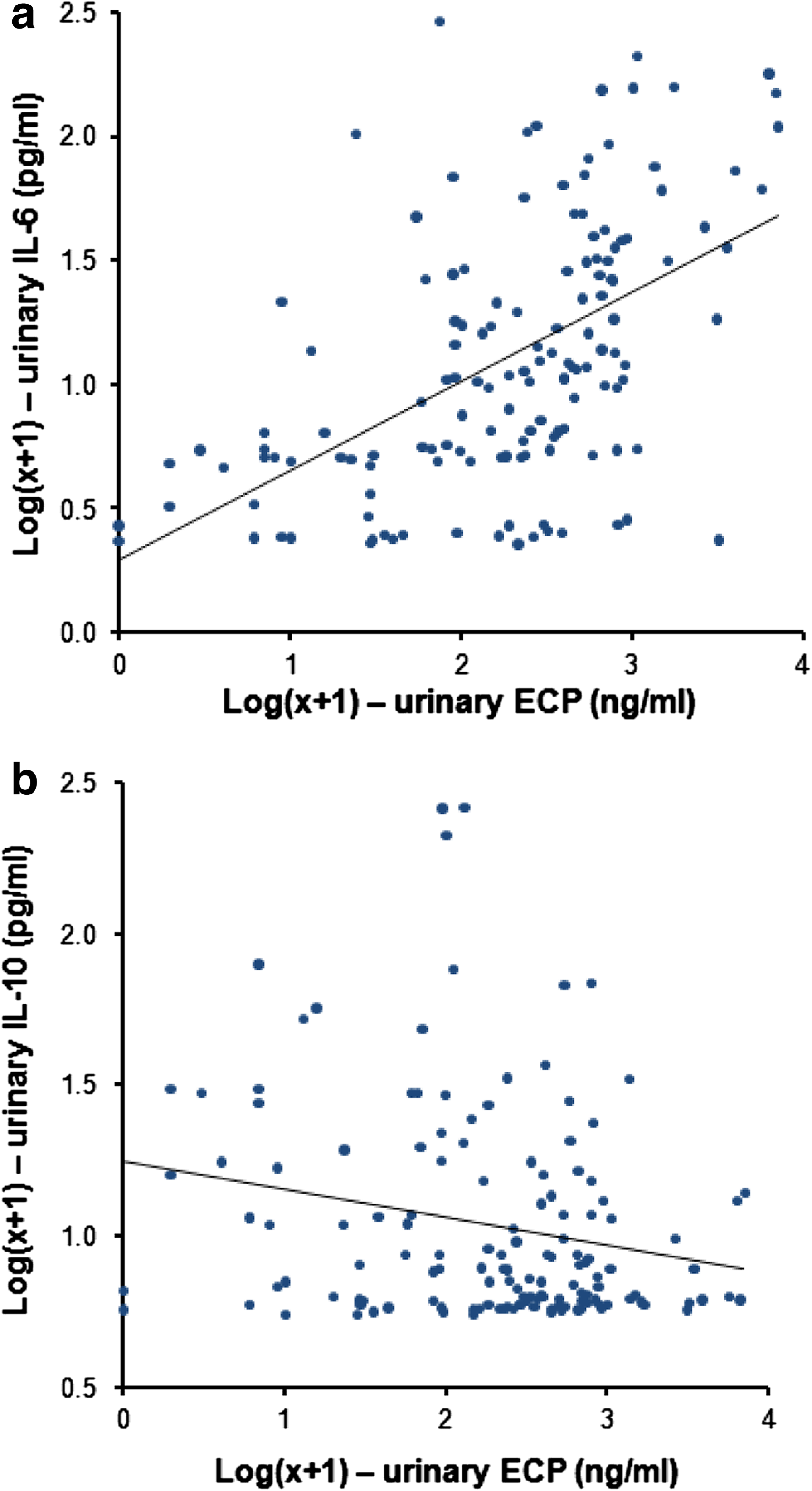

Supplement: Supplementary file 3 — Authors’ original file for figure 3 [file 12879_2014_3829_MOESM3_ESM.tif]
